# The impact of vitamin D supplementation as an adjuvant therapy on clinical outcomes in patients with severe atopic dermatitis: A randomized controlled trial

**DOI:** 10.1002/prp2.679

**Published:** 2020-11-03

**Authors:** Noha O. Mansour, Amal Ahmed Mohamed, Maha Hussein, Eman Eldemiry, Aliaa Daifalla, Soha Hassanin, Nourelhuda Nassar, Doaa Ghaith, Eman Mohamed Salah

**Affiliations:** ^1^ Pharmacy Practice Department Faculty of Pharmacy Mansoura University Mansoura Egypt; ^2^ Biochemistry Department National Hepatology and Tropical Medicine Research Institute Cairo Egypt; ^3^ Department of Dermatology and Andrology National Research Centre Cairo Egypt; ^4^ Faculty of Pharmacy Fellow of Clinical Pharmacology Cairo University Hospitals Giza Egypt; ^5^ Department of Dermatology, Venerology, and Andrology Faculty of Medicine Benha University Benha Egypt; ^6^ Biochemistry Department Faculty of Pharmacy Modern University for Technology and Information Cairo Egypt; ^7^ Clinical Pathology Department Elsahel Teaching Hospital Cairo Egypt; ^8^ Clinical and Chemical Pathology Department Faculty of Medicine Cairo University Giza Egypt; ^9^ Department of Dermatology, Andrology, Sexual Medicine and STDs Faculty of Medicine Helwan University Cairo Egypt

**Keywords:** atopic dermatitis, Eczema, severe, vitamin D

## Abstract

Vitamin D supplementation with standard treatment yielded positive clinical outcomes in mild and moderate atopic dermatitis; however, the potential benefit of vitamin D in severe cases remains unclear. This study aimed to evaluate the impact of vitamin D supplementation on response to standard treatment in pediatrics with severe atopic dermatitis. The patients were randomized to receive either vitamin D 3 1600 IU/day or placebo, plus baseline therapy of topical 1% hydrocortisone cream twice daily for 12 weeks. The primary endpoints were the change in mean Eczema Area and Severity Index (EASI) score at the end of the study and the mean percent change in EASI score from baseline to week 12. Eighty‐six subjects completed the study. The treated group achieved a significant higher level of 25 hydroxy vitamin D (*P* < .001) compared to control group at week 12. The mean EASI score was significantly lower in the treatment group compared to placebo group (*P* = .035). The percent change in EASI score from baseline differed significantly between the supplementation (56.44 ± 29.33) and placebo (42.09 ± 19.22) groups after intervention (*P* = .039). Vitamin D supplementation could be an effective adjuvant treatment that improves the clinical outcomes in severe atopic dermatitis.

## Graphical abstract

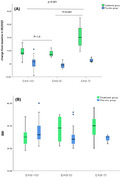

**Abbreviations**
AD: Atopic dermatitis
EASI: Eczema Area and Severity Index
NHTMRI: National Hepatology and Tropical Medicine Research Institute
Vit D: vitamin D

## 1 INTRODUCTION

Atopic dermatitis (AD) is a chronic relapsing inflammatory skin disease with intermittent flares and debilitating effects on the patient's quality of life. It is the most common skin disorder in children, affecting approximately 15% to 20% worldwide.^1^ Atopic dermatitis is clinically distinguished by pruritus, eczematous plaques, and a defective epidermal barrier.^2^ The pathology of AD is not entirely understood. It involves a complex interplay of dysfunctions of immune response, genetic and environmental factors.^3^ Currently, the conventional AD treatments include immune modulatory agents, such as topical and/or oral steroids and topical calcineurin inhibitors.^4^ The control of patients with AD may be difficult to be achieved in some patients; this suggests the presence of some other associated factors. The findings obtained in both clinical and observational studies revealed that the deficiency of vitamin D (Vit D) may be a factor to be considered in the pathophysiology of AD.^5^

Vitamin D3 correlate well with synthesis of proteins that are necessary for skin barrier function, these mechanisms suggest a role of 1,25‐dihydroxyvitamin D in modulating AD severity.^8^ Many researches have investigated difference between 25‐dihydroxyvitamin D 25(OH) D levels in AD pediatric patients and matched healthy control. A meta‐analysis of these studies found a mean deference of −16 nmol/L in pediatric AD patients compared to healthy control.^6^ There is growing interest in the possible role of vit D deficiency in the development of AD. The aggravation of AD in winter, especially in higher‐latitude countries, where serum 25(OH)D levels tend to be predominantly low in this season, has been documented.^7^ In addition, genetic polymorphisms of the Vit D receptor have been identified as contributor to the development of AD.^8^

A recent meta‐analysis of interventional studies documented that Vit D supplementation was linked to clinically relevant reduction in AD disease severity both in adult and pediatric patients.^6^ The results of this analysis must be interpreted with caution particularly for children due to presence of multiple serious limitations. First, the analysis included only one randomized controlled trial with very limited sample size (n = 20) in the age group from 1 to 18 years old.^9^ Another notable limitation is that the AD patient population involved in this analysis consisted mostly of mild and moderate AD with very few severe cases.^9^, ^10^, ^11^ Therefore, the results could not be generalizable to pediatric patients with severe AD who is limited yet important subset of patient population.

To the best of our knowledge, this is the first study to investigate potential benefits of Vit D supplementation in children and adolescents with severe AD. Therefore, the primary aim of this trial was to determine the impact of Vit D supplementation in conjunction with standard treatment in severe AD.

## 2 METHODS

### 2.1 Study design

This study was a double blind, randomized, parallel, placebo controlled clinical trial performed at the National Hepatology and Tropical Medicine Research Institute (NHTMRI), Cairo, Egypt. The study was approved by NHTMRI research ethical committee. The protocol was registered under the identifier NCT04468711. The trial was conducted in accordance with Good Clinical Practice guidelines and the Declaration of Helsinki. Informed consent was obtained from the parents of all cases.

### 2.2 Subjects

Subjects enrolled in the period from 6th June to 1th September, 2018. Inclusion criteria included: patients aged from 5 to 16 years old, with a diagnosis of severe AD according to Hanifin and Rajka criteria, and the Eczema Area and Severity Index (EASI) score.^12^ Reasons for exclusion were serious skin disorder other than AD, taking systemic corticosteroids or anti‐inflammatory medications, prior vitamin D supplementation, receiving oral or topical antibiotics or topical calcineurin inhibitors for at least 1 week prior to enrolment, known gut absorption problem, presence of active skin infection at baseline, and any known hepatic and/or renal disease.

Participants were allocated in 1:1 ratio to receive either vitamin D_3_ 1600 IU/day or placebo group, plus baseline therapy of topical 1% hydrocortisone cream twice daily for 3 months. We used a computer random number generator to form the allocation list for the two comparison groups. Treatment allocation was concealed in sequentially numbered, sealed, opaque envelopes from the patients, and the outcome assessors. The upper tolerable limit, defined as the highest level of daily vitamin D_3_ intake that is safe in the general population, for vitamin D_3_ is 3000 IU/d in children ages 4‐8 years, and 4000 IU/d in adolescents and adults.^13^ Data from clinical trials indicated that daily supplementation with this dose (1600/d) result in a clinically meaningful AD severity reduction.^6^ We assumed that this dose would be safe and effective as well. Treatment assignment was masked from the participants and the investigators. A dietary history was obtained at study entry with attention to potential sources of vitamin D, no significant group differences were prominent, and diets were stable during the study. A single pediatric dermatologist performed all clinical evaluations at baseline and at the end of the study. At baseline patient demographic data, laboratory analysis and clinical characteristics were collected.

### 2.3 Serum 25(OH)D analysis

Two milliliters of blood were withdrawn from patients, allowed to clot, and then centrifuged for 10 minutes and then kept frozen at −80°C at the Central Labs of NHTMRI, Cairo, Egypt. Quantitative determination of serum 25(OH) D, using commercial automated ELISA, DRG International Inc, USA, according to manufacture instructions, was performed.^14^ For the primary analysis in this study, we categorized the serum 25(OH)D levels into three clinically relevant ranks identified by the Endocrinology Society Clinical Practice Guidelines^15^ which are deficient (<20 ng/mL), insufficient (21‐29 ng/mL), and sufficient (>30 ng/mL).

### 2.4 Clinical assessment

Dermatological examination was performed to all the patients to assess the dermatitis severity using EASI score.^16^ It is a tool used to evaluate the severity of eczema in four defined body regions (head and neck, torso, arms, and legs), evaluating severity of four clinical signs (erythema, induration/papulation, excoriation, and lichenification) on a 4‐point scale and weights these factors based on the size of the anatomic area being evaluated. Extent is measured from 0 (0% involvement) to 6 (90%‐100% involvement), and severity is measured from 0 (clear) to 3 (severe) for each sign. This provides a range of EASI scores from 0 to a maximum score of 72. The potential severity strata for EASI is 0 almost clear, 0.1‐1 clear, 1.1‐7 mild, 7.1‐21 moderate, 21.1‐50 severe, 50‐72 very severe.^12^ Patients were clinically evaluated every 4 weeks.

### 2.5 Outcomes

#### 2.5.1 Primary endpoints

The change in mean EASI score at the end of the study and average percent change in EASI score from baseline to week 12.

#### 2.5.2 Secondary end points

Included proportion of patients with a reduction from baseline to week 12 of:

- ≥75% on EASI score (EASI 75).
- ≥50% to <75% on EASI score (EASI 50).
- < 50% on EASI score (EASI <50).

### 2.6 Statistical analysis

#### 2.6.1 Sample size

Considering the reduction in disease severity after Vit D supplementation reported by Sanchez‐Armendariz et al,^4^ a sample size of 84 patients was needed to provide at least 80% power and a two‐sided type I error less than 0.05. The sample size was calculated using the G*Power© software (Institutfür Experimentelle Psychologie, Heinrich Heine Universität, Düsseldorf, Germany) version 3.1.9.2.

Statistical analysis was done using IBM SPSS^®^ Statistics version 22 (IBM^®^ Corp., Armonk, NY, USA). Numerical data were expressed as mean and standard deviation or median and range as appropriate. Qualitative data were expressed as frequency and percentage. Numeric data were tested for normality using Shapiro‐Wilk test. Data were found not normally distributed, so the nonparametric tests were used. Comparison between two groups was done using Mann‐Whitney test (non‐parametric *t*‐test). Comparison between 3 groups was done using Kruskal‐Wallis test (non‐parametric ANOVA) then post‐Hoc test was used for pair‐wise comparison based on Kruskal‐Wallis distribution. Spearman‐rho method was used to test correlation between numerical variables. Wilcoxon‐signed ranks test (non‐parametric paired *t*‐test) was used to compare two consecutive measures of numerical variables.

## 3 RESULTS

### 3.1 Participant characteristics

**FIGURE 1:**
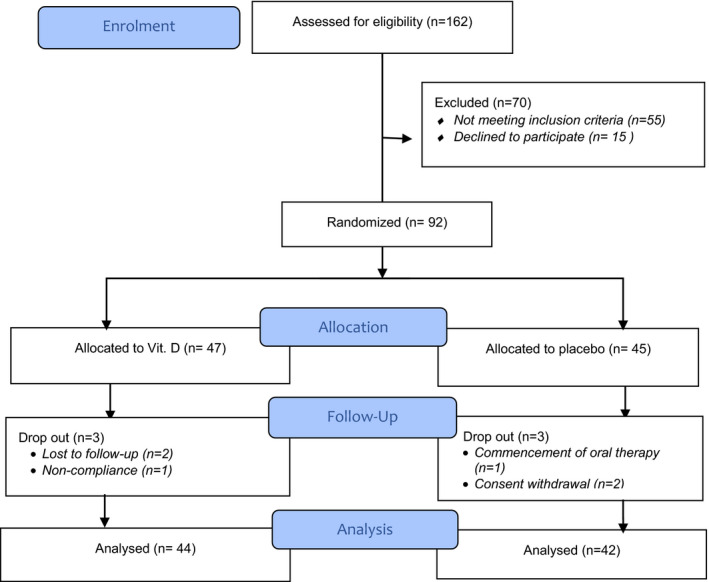
CONSORT flow diagram showing the flow of patients throughout the study

As shown in Figure 1, 95% (n = 86) of the randomized subjects completed the study and was included in the final analysis. At baseline, both groups were comparable in demographic and clinical characteristics. Patients’ demographics and clinical characteristics at baseline were summarized in Table 1.

**TABLE 1:** Baseline demographics and clinical characteristics for both groups

	Treatment group		Placebo group	*P* value
Age (years)^a^	12 (4.75)		11 (5.5)	.06
Gender; n (%)				.13
Male	26 (59.1)		18 (42.8)	
Female	18 (40.9)		24 (57.1)	
BMI (kg/m^2^)	27.1 (5.3)		26.6 (4.7)	
BMI categories				.20
Normal weight n (%)	15 (34.1)		12 (28.6)	
Overweight n (%)	13 (29.5)		20 (47.6)	
Obese n (%)	16 (36.4)		10 (23.8)	
Serum 25(OH) D levels	22.8 (6.2)		25.4(8.1)	.18
Categories; n (%)				.34
<20 ng/mL (deficient)	15 (34.1)		11 (26.1)	
20‐29 ng/mL (insufficient)	22 (50)		19 (45.2)	
≥30 ng/mL (sufficient)	7 (15.9)		12 (28.6)	
EASI score	44.4 (6.28)		46.4 (5.4)	.10
Calcium (mg/mL)	8.81(0.87)		8.7(1.03)	.49
Parathyroid hormone (pg/mL)		32.4(5.7)	32.1(6.7)	.88

Abbreviations: BMI, body mass index; EASI, Eczema Area and Severity Index.

^a^ Median (IQR).

### 3.2 Serum 25 (OH) D concentrations

At base line, no statistically significant difference was found between both study arms regarding the 25(OH) D serum levels (*P* = .18). In addition, distribution of 25(OH)D deficiency categories was similar between the two groups (Table 1). Association between baseline 25(OH) D levels and potential deficiency risk factors was explored in the whole study subjects. Inverse weak relationship was established between base line 25(OH) D serum levels and the body mass index (BMI) (Spearman's rho *r* = −.44, *P* < .001). Weak association was registered between baseline 25(OH)D serum levels and initial EASI score (Spearman's rho *r* = .34, *P* = .001).

In the Vit D group, significant improvement in 25(OH) D serum levels was achieved postsupplementation compared to baseline (*P* = <.001). Ninety‐three percent (n = 4) of the vitamin D group population reached sufficiency level (>30 ng/mL). The maximum serum 25(OH) D reached in this group was 50 ng/mL, concentration below which toxicity has not been observed.^17^

In the placebo group, level of 25(OH) D was comparable to baseline level (*P* = .47) at the end of the study. At week 12, about 74% of the placebo group subjects remained under levels of sufficiency (<30 ng/mL). Significantly higher level was recorded between supplemented group (36.11 ± 5.84) and placebo group (25.86 ± 8.27) at the end of the study regarding serum 25(OH) D levels (*P* < .001).

**FIGURE 2:**
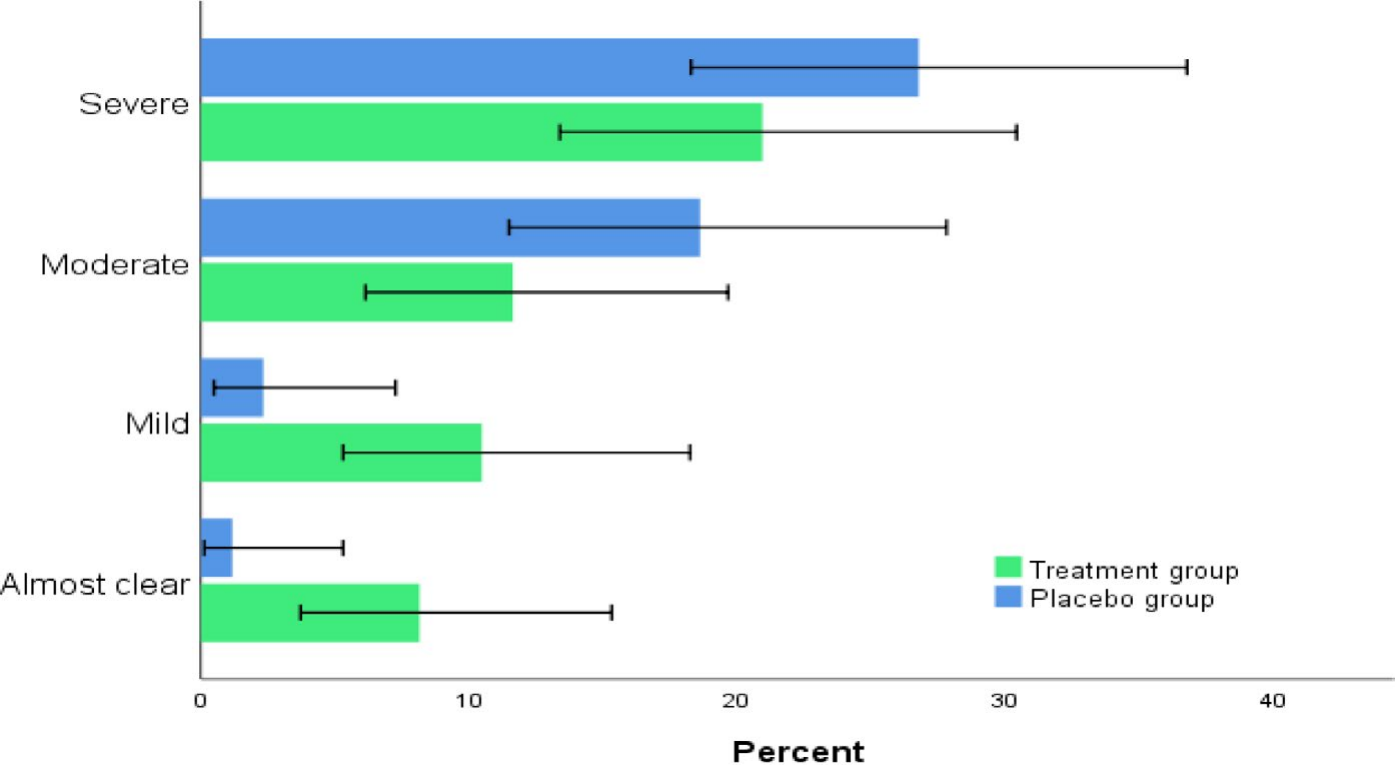
Severity of AD at the end of the study for both groups. Error bars: 95% CI, (*P* < .05)

**FIGURE 3:**
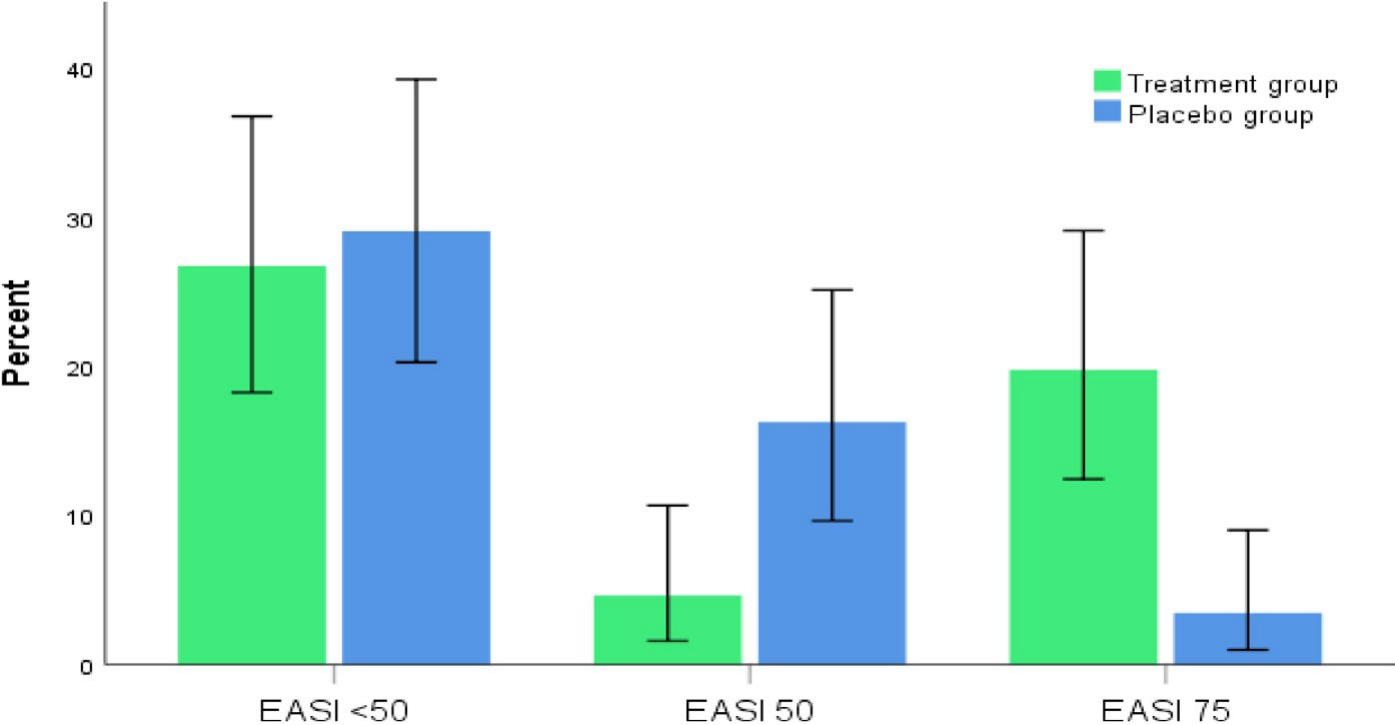
Percentage of patients who achieved <50% improvement in EASI score (non‐responders), achieved ≥50% to <75% improvement (EASI 50), achieved ≥75% improvement (EASI 75) at the end of the study in both groups. Error bars: 95% CI, (*P* < .05)

Table 2 showed that children supplemented with vit D fared better than those allocated to placebo. At the end of the study, the mean percentage change from baseline in EASI score was significantly greater with vitamin D group (56.44%) than with placebo group (42.09%) (*P* = .039). Figure 2 depicts the different response category attained at the end of the study. Figure 3 showed that comparable proportion in the vitamin D group and placebo group (52.2% vs 59.5%) experienced modest response to treatment (EASI < 50). On contrast, different patterns were notable between supplemented patients and those allocated to placebo group regarding percentage of patients who achieved EASI 50 or EASI 75. Notably, about 38.6% of supplemented patients achieved EASI 75 vs only 7.1% of patients in the placebo group.

**TABLE 2:** Change in severity of AD and serum 25 (OD) D levels for both groups at the end of the study

	Treatment group	Placebo group	*P* value
Mean EASI score	20.42 (14.6)	27.47 (10.11)	.035
% change in EASI from baseline	56.44 (29.33)	42.09 (19.22)	.039
Serum 25(OD) D levels	36.11 (5.84)	25.86 (8.27)	<.001
Categories n (%)			<.001
<20 ng/mL (deficient)	0 (0)	8 (19.04)	
20‐29 ng/mL (insufficient)	3 (6.81)	23 (54.76)	
≥30 ng/mL (sufficient)	4 (93.18)	11 (26.19)	

**FIGURE 4:**
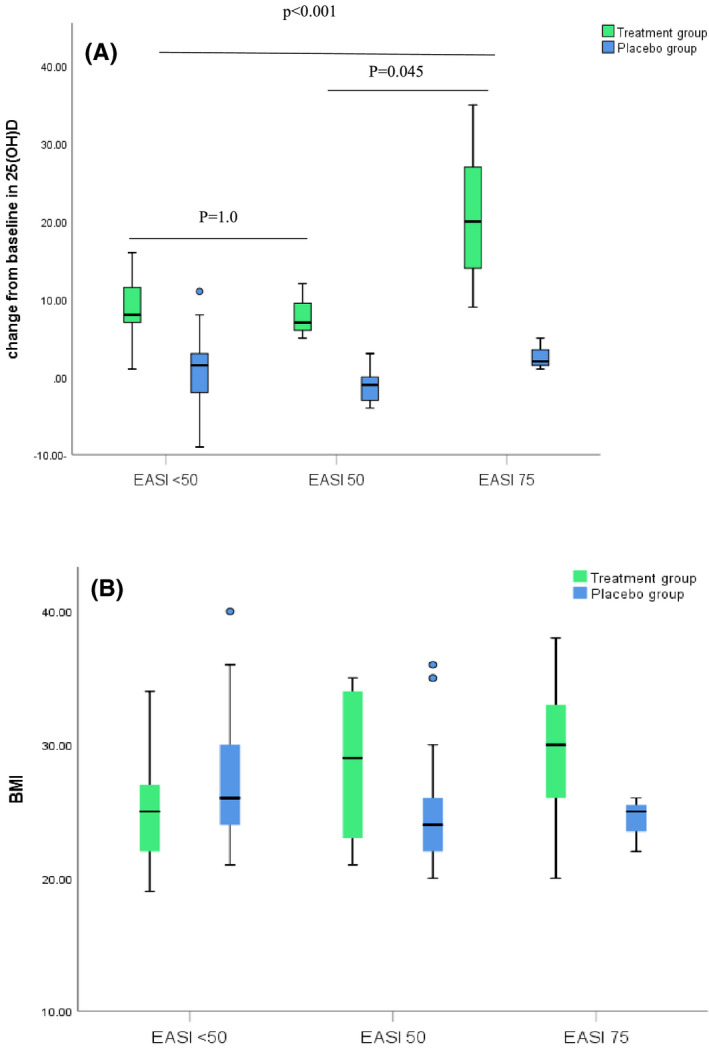
Clustered boxplot showing (A) the distribution of change in vitamin D levels [*P* value < .001 (treatment group), =.294 (placebo group)] (B) the distribution of BMI [*P* value = .057 (treatment group), =.197 (placebo group)] among patients who achieved EASI < 50, EASI 50 and EASI 75

Potential predictors that might cause superior clinical outcomes among patients who achieved EASI 75 were further investigated. Percent change in EASI score significantly correlated with the magnitude of change from baseline in 25(OH)D (Spearman's rho *r* = .6, *P* = .005). However, fair correlation was established between BMI and % change in EASI score (Spearman's rho *r* = .54, *P* = .01) in this subset of patients, which might indicate causality. To test the hypothesis that different magnitude of change from baseline in serum 25(OH)D exists among patients with better response category, the non‐parametric Kruskal‐Wallis test in combination with pairwise post‐hoc test was performed to compare the respective significant group; results presented in Figure 4. Pairwise comparisons revealed a significant relationship between both EASI < 50 and EASI 75 (*P* < .001) and between EASI 50 and EASI 75 responders (*P* < .001) groups. Regarding BMI, the results did not reach statistical significance when the different response categories were compared regarding distribution of BMI among different respondents’ ranks. (*P* = .057).

## 4 DISCUSSION

Standard initial treatment modalities for the management of AD are centered around the use of topical steroid preparations and moisturization of the skin. Patients with severe disease who fails to improve with this initial conventional therapy might benefit from second‐line therapies, such as systemic and topical immunosuppressive medications.^18^ Most of these therapies have potential adverse effects and nearly all are off label for AD in children. The present study was designed to test the hypothesis that vitamin D supplementation as an adjuvant therapy might benefit the severe AD children, and since recent evidence has demonstrated that it improved the clinical outcome in mild and moderate AD pediatric patients.

According to our knowledge our study is the first to assess the efficacy of vit D in conjunction with standard treatment in patients with severe eczema. At baseline, there was no significant difference between the two groups in serum level of 25(OH)D. High prevalence of 25(OH)D deficiency was notable among all study population. Similar finding has been previously reported in Egyptian children with AD and in healthy control as well.^3^ To understand the 25(OH)D status associated with AD, the factors that might influence 25(OH)D deficiency were investigated. In the present study, lower baseline 25(OH) D levels were observed in obese patients. Some trials reported similar inverse relationship,^19^ while others not.^20^ This negative influence of obesity has been suggested to be due to the lipophilic nature of Vit D and distribution into the increased stored fat in subjects with high BMI.^19^

At the end of the study, a statistically significant difference was found between both study arms regarding the mean EASI score, and the mean % change from their baseline. The impact of Vit D oral supplementation as an adjuvant therapy on eczema severity modification has been previously investigated. In line with the results of the present study, Oral vit D supplement reduced the skin colonization of *S aureus* and demonstrated clinical improvement in children with moderate eczema.^9^ Similarly, oral Vit D supplementation has been shown to improve winter‐related AD symptoms.^21^ The observed improvement in disease severity from vitamin D supplementation has strong biological plausibility as 1,25 (OH)D contributes to hallmark features of AD: altered barrier function, immune dysregulation, and inadequate bacterial defense. This might explain the positive impact of supplementation recorded in the present study. Opposing our finding, Galli et al^22^ reported that daily oral Vit D_3_ supplementation for 3 months do not correlate with the severity of chronic eczema in children. Lack of correlation with our results might be attributed to the difference in the patient population as the majority (53.9%) of their enrolled children had 25(OH)D sufficiency at baseline and 74% presented with mild eczema. Likewise, Sidbury et al^23^ demonstrated in a pilot study that Vit D supplementation did not significantly influence the severity of disease in children. The small sample size (n = 12) and short duration of vit D supplementation^1^ might explain this lack of connection with our results.

The EASI score was chosen by the international Harmonizing Outcomes Measures in Eczema group (HOME) to be included as a core clinical outcome measure in AD clinical trials.^24^ Validation studies confirm that the EASI score has adequate reliability, validity, and responsiveness which represent the key performance properties needed for any outcome instrument. However, data regarding how a clinician would interpret an EASI score into clinically meaningful information are not available.^25^ It can be seen from checking the banding of different EASI strata that the distribution of severity scale across strata is not equal. This skewness makes changes in the lower end of the score more clinically important than changes in the upper end. There are no previous reports that clearly define a responder threshold for % change from baseline in EASI score for patients with severe AD; however, stratifying patients according to % reduction in EASI scores to EASI 50 and EASI 75 was considered by many pivotal trials to illustrate clinically important differences.^26^, ^27^, ^28^ At the end of the study, significant difference between the two groups was obtained regarding proportion of patients achieving EASI < 50, EASI 50, and EASI 75 (*P* < .001). In treatment group, 38.6% vs 7.1% in control group achieved EASI 75. However, the percentage of non‐responders deemed comparable between the supplemented patients and those allocated to placebo (59.5% vs 52.2%, respectively). This indicates that some supplemented patients might achieve excessive benefit from treatment. Indeed, diverse factors could be linked to this preferential response. On one hand, fair correlation between BMI and % change in score was established among EASI 75 respondents. Moreover, 25(OH)D deficiency was high prevalent among overweight and obese patients at the beginning of the study. Since the baseline 25(OH)D deficiency has been previously shown to alter response to supplementation in adults^29^ and adolescents.^30^ So, it is conceivable to suggest that the high % reduction in EASI score postsupplementation in some obese patients might be influenced by the baseline 25(OH) D concentration; however, the distribution of BMI was not statistically different among different response categories. Further studies are needed to elucidate whether response to supplementation would vary according to the BMI in patient with severe eczema.

On the other hand, statistically significant difference existed between the different response categories and magnitude of change in 25(OH)D serum level. This finding might be illustrated in different ways. Firstly, variation in the factors that negotiate absorption efficiency of oral supplementation in the gastrointestinal tract (GIT) might have existed among some supplemented patients. These factors include variations in the amount and type of fatty acids,^31^, ^32^ dietary fibers, and the interaction with other fat soluble micronutrients.^33^ Second, the host‐associated factors such as genetic variation might provide another explanation.^34^ Thus, it is plausible to hypothesize that the bioavailability of vit D in GIT is compromised in some patients due to variation within these previously mentioned factors, however, a clear cut is yet lacking.

One limitation of our study is that the study population was comprised of patients with limited ethnic diversity, potentially restricting its generalizability. Future studies on more diverse populations are needed. Another limitation, lack of data from other important domains, such as patient reported outcomes. Moreover, given the possible seasonal fluctuations that characterize AD, future trials are needed to determine if the benefits of supplementation would sustain in patients with winter‐related severe eczema.

In conclusion, our study suggests that oral daily Vit D supplement might provide clinical improvement in children with severe AD. More investigations are needed to reveal factors associated with superior clinical outcomes in some supplemented patients. We advocate further multicenter studies with larger sample size of ethnic diverse population to validate the potential benefit of vit D on clinical outcomes of severe pediatric eczema. Further studies are also needed to examine whether the positive impact of supplementation would be maintained in pediatrics with winter‐related severe eczema.

## AUTHOR CONTRIBUTIONS

Participated in research design: Eman A., Amal, Noha, and Maha. Conducted the clinical part: Eman A, Aliaa, Maha, Eman E., Soha, Nourelhuda, and Doaa. Contributed to the laboratory analysis: Amal, Nourelhuda, and Doaa. Performed data analysis: Noha, Eman, and Soha, Eman E. Drafted the manuscript: Maha, Nourelhuda, Doaa, and Noha. Edited and submitted the final manuscript: Eman A., Amal, and Noha.

## DATA SHARING AND ACCESSIBILITY

The data that support the findings of this study are available from the corresponding author upon reasonable request.
